# Iron Deficiency Inhibits the Proliferation of Intestinal Stem Cells and Induces Their Differentiation to Enterocytes

**DOI:** 10.3390/nu18030392

**Published:** 2026-01-24

**Authors:** Yecheng Xu, Jing Zhao, Shouchuan Jiang, Yu Han, Yi Zheng, Xi Qiao, Xin Wen, Yuanyuan Zhang, Yunqin Li, Jingxia Kong, Huahua Du

**Affiliations:** 1 Zhejiang Key Laboratory of Nutrition and Breeding for High-Quality Animal Products, College of Animal Sciences, Zhejiang University, Hangzhou 310058, China; 2 Analysis Center of Agrobiology and Environmental Science, Zhejiang University, Hangzhou 310058, China; 3 Shulan International Medical College, Zhejiang Shuren University, Hangzhou 310015, China

**Keywords:** iron deficiency, intestinal stem cell, enteroid, proliferation, differentiation

## Abstract

**Objectives**: Iron deficiency impairs intestinal mucosal structure and function, yet its impact on intestinal stem cells (ISCs) remains unclear. This study was therefore designed to examine how iron deficiency affects the proliferation and differentiation of ISCs. **Methods**: Iron-deficient mouse and enteroid models were established. Expression of key cell markers was analyzed using Western blot, qPCR, and immunofluorescence. **Results**: Iron deficiency led to structural impairment of the intestinal mucosa, characterized by decreased small intestinal villus height. In iron-deficient mice, expression of ChrA (enteroendocrine cell marker), Lyz (Paneth cell marker), and Muc2 (goblet cell marker) was significantly downregulated across duodenum, jejunum and ileum, whereas Vil1 (enterocyte marker) expression increased. Moreover, both Lgr5 (an ISC marker) expression and the number of Ki67-positive proliferating cells were significantly reduced, along with a decrease in Ki67 transcriptional levels under iron-deficient conditions. Similarly, deferoxamine (DFO)-treated enteroids showed fewer Lgr5-positive ISCs, downregulation of Lgr5, Lyz and Muc2, and upregulation of Vil1. RNA-seq further confirmed that iron deficiency skews ISC differentiation toward absorptive lineage. This shift was associated with modulation of the Notch signaling pathway: upregulation of the ligand *Dll1*, receptors *Notch2* and *Notch3*, and the protease *ADAM10*, alongside downregulation of the negative regulator *Atoh1*. These findings indicate that Notch pathway activation promotes enterocyte differentiation under iron deprivation. **Conclusions**: Iron deficiency suppressed the proliferation of ISCs and induced their differentiation toward enterocytes, which is associated with the modulation of the Notch signaling pathway, providing a mechanistic insights for impaired intestinal repair and the potential for nutrient-targeted therapies.

## 1. Introduction

Iron deficiency represents a significant global public health challenge, affecting approximately one-third of the world’s population [1]. Its consequences extend far beyond the classic hematological manifestations of anemia, impacting multiple organ systems [2].

Within the gastrointestinal tract, iron deficiency is clinically associated with a spectrum of structural and functional impairments, most notably villous atrophy, malabsorption, and intestinal barrier dysfunction [3]. These alterations not only reduce quality of life but also create a pernicious cycle in which impaired gut function further impedes nutrient absorption, aggravating the underlying deficiency [4]. Although these clinical outcomes are well established, the fundamental cellular and molecular mechanisms driving this intestinal pathology remain inadequately defined, representing a critical obstacle to the development of targeted therapeutic strategies.

At the heart of intestinal resilience lies the dynamic population of intestinal stem cells (ISCs), residing within the crypts of Lieberkühn. These cells serve as the primary architects of epithelial renewal, responsible for the continuous and high-fidelity regeneration of the entire intestinal lining [5]. Through precisely orchestrated divisions, ISCs give rise to transient amplifying cells, which subsequently differentiate into the diverse, functional epithelial lineages of the epithelium: nutrient-absorbing enterocytes and various secretory cells, including mucus-producing goblet cells, antimicrobial Paneth cells, and hormone-secreting enteroendocrine cells. This exquisite balance between ISC self-renewal and lineage commitment is the cornerstone of mucosal homeostasis [6,7]. Crucially, the maintenance of this stem cell compartment is both energetically and biochemically demanding.

Iron serves as an essential cofactor for numerous proteins involved in critical biological processes, including mitochondrial electron transport, oxidative phosphorylation, and DNA synthesis, making it a fundamental metabolic currency [8]. The emerging recognition that a majority of functional ISCs operate in an iron-sensitive metabolic state underscores their potential susceptibility to fluctuations in iron bioavailability [9]. This intrinsic dependency positions iron availability as a likely pivotal regulator of ISC behavior, influencing their fate decisions between proliferation, quiescence, and differentiation [10]. However, a direct, mechanistic link between systemic iron deficiency and the autonomous reprogramming of ISC biology has not been conclusively established.

To address this critical knowledge gap, our study aims to systematically investigate the hypothesis that iron deficiency directly impairs intestinal homeostasis by reprogramming the metabolism of ISCs and biasing their differentiation commitment. We will employ an integrated experimental approach that combines a physiologically relevant in vivo mouse model of dietary iron deficiency with a controlled in vitro intestinal organoid system. This strategy will allow us to dissect the cell-autonomous effects of iron scarcity on ISCs, independent of systemic secondary influences. Our primary objectives are to (1) quantitatively evaluate the impact of iron deficiency on ISC proliferation and pool maintenance; (2) define alterations in lineage specification under iron-restricted conditions; and (3) identify key signaling pathways and metabolic shifts that mediate the ISC response to iron deficiency.

## 2. Materials and Methods

### 2.1. The Animals

Three-week-old male C57BL/6 mice were purchased from Charles River Laboratories (Beijing, China). All mice were housed under specific pathogen-free (SPF) conditions at Zhejiang University and had free access to water and appropriate diets. A chronic model of dietary iron deficiency was generated by feeding 3-week-old mice either a low-iron diet (5 mg/kg Fe, *n* = 8) or a standard diet (150 mg/kg Fe, *n* = 8) for 60 days. Both the low-iron diet and standard diet, formulated to be identical in all nutritional components except for iron content, were purchased from Xietong Biotechnology (Nanjing, China). At the conclusion of the experiment, blood samples were collected by retro-orbital puncture. The mice were then euthanized via cervical dislocation, after which intestinal tissues were rapidly frozen in liquid nitrogen and stored at −80 °C for subsequent analysis. Additionally, enteroids derived from intestinal crypts of 8-week-old mice were treated for 48 h under three conditions: control (culture medium), 500 μM deferoxamine (DFO, iron chelation group, *n* = 6), and 500 μM DFO + 500 μM ferric ammonium citrate (FAC, iron repletion group, *n* = 6), establishing an in vitro intervention model to resolve cellular iron compensation mechanisms. The procedures were approved by Zhejiang University Animal Care Committee (ZJU20240918).

### 2.2. Measurement of Serum Indicators

The serum iron concentration was measured using a commercial assay kit (Iron Assay Kit, Elabscience Biotechnology, Wuhan, China; Catalog No. E-BC-K945-M) based on the principle of ferrous iron reacting with ferrozine to form a purple complex, with absorbance detected at 562 nm. Total iron binding capacity was measured using a specialized test kit (Total Iron Binding Capacity Assay Kit, Jiancheng, Nanjing, China; Catalog No. A040-1-1) following the chromogenic principle of iron-binding protein combining with iron ions. Absorbance readings were taken at 520 nm with a microplate reader (Molecular Devices, Sunnyvale, CA, USA). Transferrin saturation was calculated as follows: (serum iron [mg/L]/total iron binding capacity [mg/L]) 100. Hemoglobin concentration in peripheral blood was measured using an automated hematology analyzer (XN-1000V, Sysmex, Hamburg, Germany) following the manufacturer’s standard procedures for erythrocyte parameter detection.

### 2.3. RNA Sequencing and Analysis

We used total RNA with a minimum amount of 800 ng to create strand-specific libraries. This process involved several steps: poly-A selection, cDNA fragmentation, and adapter ligation. We quantified the resulting libraries using a Qubit 3.0 fluorometer and validated their size with a Qsep100 system. Then, we sequenced them on the NovaSeq 6000 platform using a 150 bp paired-end approach. We rigorously filtered the raw sequencing data, applying Q20 and Q30 thresholds and performing adapter trimming. Next, we used featureCounts to count reads and then quantified gene expression as FPKM. Differential expression analysis was conducted using DESeq2. Using biological triplicates, we set the criteria for differential expression as |log_2_FC| greater than 1 and a *p*-value less than 0.05. Gene Ontology (GO) and Kyoto Encyclopedia of Genes and Genomes (KEGG) pathway enrichment analyses were performed with the cluster Profiler package. The significance threshold was set at *p* less than 0.05. We effectively visualized the key findings using various graphical representations such as heatmaps, volcano plots, and Venn diagrams.

### 2.4. Histological Examination

Tissue specimens were fixed overnight in 4% paraformaldehyde, rinsed in phosphate-buffered saline (PBS), dehydrated through an ethanol series, and embedded in paraffin. Subsequently, 5 μm sections were deparaffinized, rehydrated, and stained with hematoxylin and eosin (H&E). Images were taken by Olympus APX100 and BX63 (Beijing, China). The villus height (from the tip of the villi to the villus-crypt junction) and crypt depth (depth of the invagination between adjacent villi) were measured and recorded using Image-Pro Plus 7.0 (Media Cybernetics, Rockville, MD, USA). For each intestinal cross-section, measurements were obtained from at least three structurally intact and properly oriented crypt–villus units to ensure accurate morphological assessment.

### 2.5. Analysis of mRNA Expression

Total RNA was extracted using the SteadyPure Universal RNA Extraction Kit (Accurate Biotechnology, Changsha, China). Subsequently, 1 μg of the isolated RNA was reverse-transcribed into cDNA with the Evo M-MLV Mix Kit with gDNA Clean for qPCR (Accurate Biotechnology, Changsha, China). Quantitative real-time PCR analysis was performed using SYBR Green Pro Taq HS Premix (Accurate Biotechnology, Changsha, China). The PCR protocol consisted of an initial denaturation at 95 °C for 30 s, followed by 40 cycles of 95 °C for 5 s and 60 °C for 30 s. Relative gene expression was calculated using the 2^−ΔΔCT^ method, with β-actin as the endogenous control. All primer sequences are listed in Table 1.

### 2.6. Western Blot Analysis

Protein extraction was performed by homogenizing cellular or murine tissue samples in RIPA lysis buffer (Beyotime Biotechnology, Shanghai, China). The buffer contained a cocktail of protease and phosphatase inhibitors (Lablead, Beijing, China). The homogenate was then clarified by centrifugation. Protein concentration was quantified with a BCA assay kit (Beyotime Biotechnology, Shanghai, China). Following SDS-PAGE separation, proteins were transferred onto PVDF membranes, which were then blocked using a rapid protein-free blocking buffer (Epizyme Biotech, Shanghai, China) and incubated overnight at 4 °C with the respective primary antibodies. Primer antibodies are listed in Table 2. Subsequently, membranes were probed with species-matched secondary antibodies for 60 min at room temperature. HRP-conjugated anti-rabbit and anti-mouse IgG (Biosharp, Hefei, China) were used as secondary antibodies. Immunoreactive bands were then visualized with Immobilon Western Chemiluminescent HRP Substrate and quantified by densitometric analysis using ImageJ software 1.54p (National Institutes of Health, Bethesda, MD, USA).

### 2.7. Isolation of Intestinal Crypts and Enteroids Culture

Intestinal organoids were generated from freshly isolated crypts taken from the small intestine as previously described [11]. The intestinal tissue was cut longitudinally and thoroughly washed before processing. Tissue segments of 5 mm length were treated with 2 mM ethylenediaminetetraacetic acid (EDTA) in PBS at 4 °C for 30 min. After EDTA treatment, the fragments were washed with ice-cold PBS. We aspirated the villus-rich supernatant and resuspended the crypt-containing pellet in fresh PBS. Mechanical dissociation was performed sequentially, followed by centrifugation, to enrich crypt structures in the supernatant. Contaminating villus debris was eliminated by 40 μm filtration (Corning, NY, USA). Purified crypts were pelleted at 200× *g* for 3 min and embedded in 50 μL Matrigel^®^ (Corning, NY, USA) in 24-well plates. Following Matrigel polymerization (37 °C, 10 min), cultures were maintained in IntestiCult™ Organoid Growth Medium (Stemcell Technologies, Vancouver, BC, Canada), with the medium replaced every 48 h. Organoids were passaged every 4–6 days.

### 2.8. Immunofluorescence (IF) Microscopy

Small intestine tissues were fixed overnight in 4% paraformaldehyde, subsequently paraffin-embedded, and sectioned at 5 µm. The sections were then deparaffinized in xylene (Google Biotech, Beijing, China) and rehydrated through a graded ethanol series. After antigen retrieval, the sections were then blocked, permeabilized, and incubated with primary antibodies and fluorescent secondary antibodies (Table 2). For immunostaining in organoids, the organoids in Matrigel were fixed in 4% PFA, then permeabilized, blocked, and incubated overnight with anti-rabbit Ki67 (1:100, Abcam, Shanghai, China), followed by incubation with anti-rabbit Alexa Fluor 594 (1:200, Abcam, Shanghai, China). Nuclei were counterstained with DAPI (1:5000, Sangon Biotech, Shanghai, China). Fluorescence images were captured using a confocal microscope (Zeiss, Jena, Germany) and an Olympus APX100 system (Beijing, China), followed by cell counting with Image-Pro Plus software IPP7.0 (Media Cybernetics, MD, USA).

### 2.9. Statistical Analysis

Statistical analysis was performed with GraphPad Prism 8.0 (GraphPad Software, San Diego, CA, USA). Data are expressed as mean ± standard error of the mean (SEM). Differences between two groups were assessed by unpaired two-tailed Student’s *t*-tests, while comparisons across multiple groups were analyzed by one-way ANOVA with Tukey’s post hoc test. A *p*-value < 0.05 was considered statistically significant, denoted as * *p* < 0.05 and ** *p* < 0.01.

## 3. Results

### 3.1. Iron Deficiency Induces Intestinal Villus Shortening and Impairs Tight Junction Proteins

**Figure 1:**
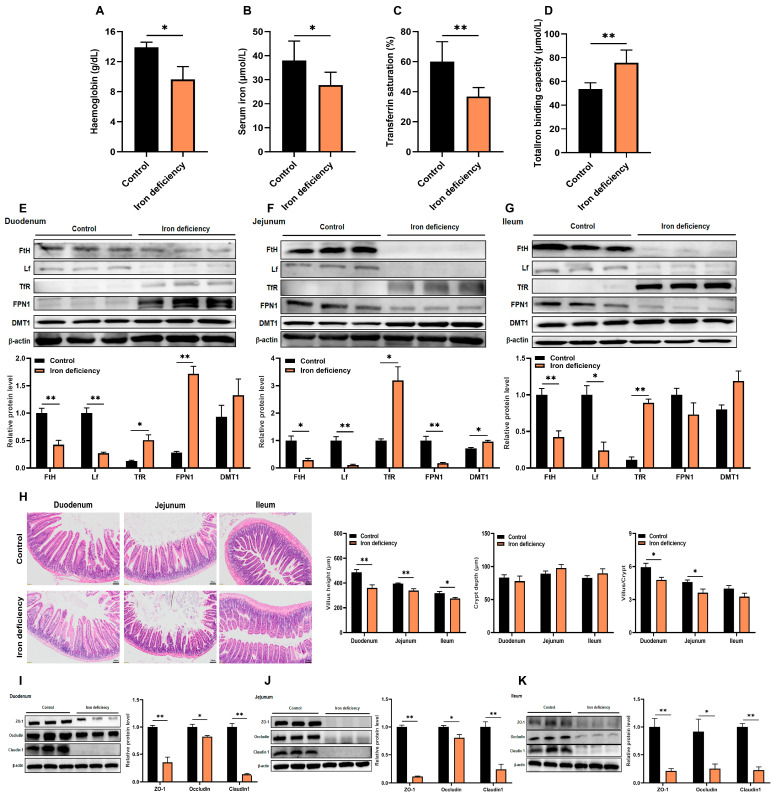
Iron deficiency induces intestinal villus shortening and impairs tight junction proteins. (**A**–**D**) Detection of hemoglobin content, serum iron level, serum transferrin saturation, and total iron-binding capacity (*n* = 5 per group); (**E**–**G**) Western blot analysis of key iron-related protein (FtH, Lf, TfR, FPN1, DMT1) expression levels in the duodenum, jejunum, and ileum (*n* = 3 per group); (**H**) Representative hematoxylin and eosin (H&E)-stained sections of the small intestine from control and iron-deficient mice (*n* = 5 per group, with 3 crypt–villus units measured per section to quantify villus height and crypt depth; Scale bar: 100 µm); (**I**–**K**) Western blot analysis of tight junction protein (ZO-1, occludin, claudin 1) expression in the duodenum, jejunum, and ileum (*n* = 3 per group). Data are presented as mean ± SEM. Statistical significance was determined using Student’s *t* test. * *p* < 0.05, ** *p* < 0.01.

In order to explore the role of iron deficiency in the intestine, we established an iron deficient murine model by feeding a low-iron diet (5 mg/kg Fe) for 60 days. Successful induction of iron deficiency was confirmed by characteristic hematological alterations: a 30.1% reduction (*p* < 0.05) in hemoglobin levels, 27.0% decrease (*p* < 0.05) in serum iron concentration, 30.1% decline (*p* < 0.01) in transferrin saturation, and a 1.4-fold increase (*p* < 0.01) in total iron binding capacity (Figure 1A–D). Western blot analysis revealed consistent iron regulatory responses across intestinal segments. In the duodenum, jejunum, and ileum of iron-deficient mice, protein expressions of ferritin heavy chain (FtH) and lactoferrin (Lf) were significantly (*p* < 0.01) downregulated, while transferrin receptor (TfR) and divalent metal transporter 1 (DMT1) were markedly (*p* < 0.05) upregulated (Figure 1E–G). Notably, ferroportin 1 (FPN1) exhibited segment-specific regulation, with significant (*p* < 0.01) upregulation in the duodenum but downregulation in the jejunum. These findings demonstrated that iron deficiency triggered adaptive increases in intestinal iron transporters (DMT1 and TfR) while suppressing iron storage proteins (FtH and Lf), collectively impairing erythropoiesis [12]. Histopathological analysis further confirmed intestinal damage. H&E staining showed significant villus shortening in iron-deficient mice: 29.9% reduction (*p* < 0.01) in the duodenum, 14.4% (*p* < 0.01) in the jejunum, and 13.4% (*p* < 0.05) in the ileum (Figure 1H). The villus height/crypt depth ratio was significantly diminished in both duodenum and jejunum (*p* < 0.05). The atrophic villi displayed structural disorganization, suggesting compromised nutrient absorption capacity and impaired mucosal barrier function. Tight junctions (TJs) are essential for epithelial barrier integrity and regulate paracellular permeability [13]. To determine whether iron deficiency affects TJ integrity, we measured the protein expression of key TJ markers (ZO-1, occludin, and claudin 1) in intestinal segments. Western blot analysis revealed that their levels were significantly reduced (*p* < 0.05) in the duodenum, jejunum, and ileum of iron-deficient mice compared with controls (Figure 1I–K), indicating that iron is critical for maintaining junctional complex integrity.

### 3.2. Iron Deficiency Inhibits the Proliferation of ISCs and Regulates Their Differentiation

**Figure 2:**
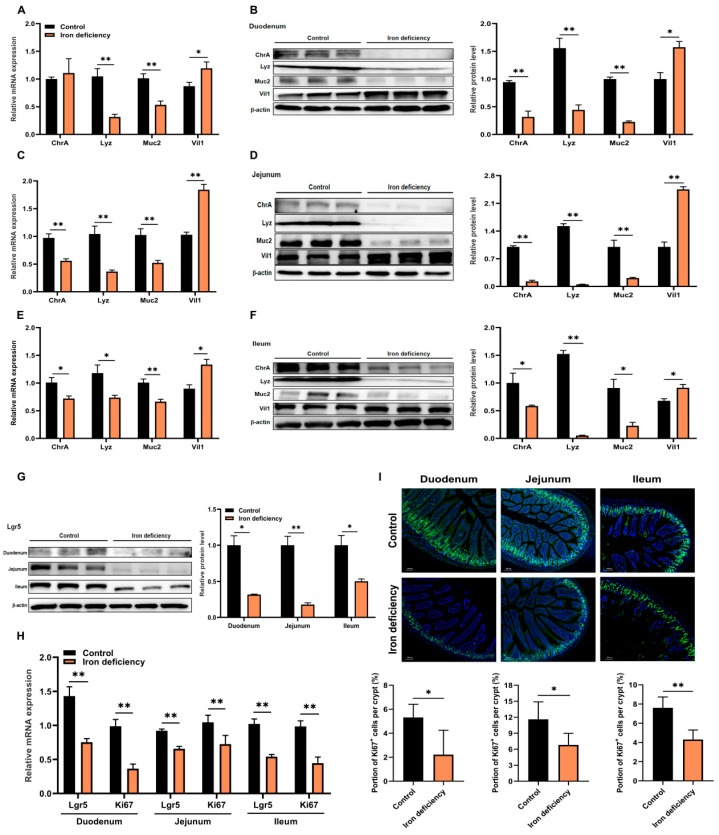
Iron deficiency inhibited the proliferation of ISCs and regulated their differentiation. (**A**–**F**) qPCR and Western blot analysis of epithelial cell markers (Vil1, Lyz, Muc2, ChrA) in the duodenum, jejunum, and ileum (*n* = 5 per group; WB data are presented as mean ± SEM from 3 technical replicates per biological sample); (**G**) Western blot analysis of Lgr5 protein expression in the duodenum, jejunum, and ileum (*n* = 3 per group); (**H**) The relative mRNA expression of Lgr5 and Ki67 in the duodenum, jejunum, and ileum (*n* = 5 per group); (**I**) Representative immunohistochemical staining sections of Ki67-positive cells in the duodenum, jejunum, and ileum (*n* = 5 per group, with 5 crypt–villus units counted per section to quantify Ki67-positive cells). DAPI, blue; Ki67-positive cells, green. Data are presented as mean ± SEM. Statistical significance was determined using Student’s *t* test. * *p* < 0.05, ** *p* < 0.01.

To further explore the impact of iron deficiency on intestinal cell fate, we examined key cell lineage markers in iron-deficient mice [14,15]. Compared with control mice, the duodenum of iron deficient mice exhibited significantly (*p* < 0.01) reduced mRNA and protein expression levels of Lyz (marker of Paneth cells) and Muc2 (marker of goblet cells), whereas Vil1 (marker of enterocytes) was significantly (*p* < 0.05) increased (Figure 2A,B). In the jejunum, iron deficiency led to a marked (*p* < 0.01) decrease in ChrA (marker of enteroendocrine cells), Lyz and Muc2 expression, while inducing Vil1 levels (Figure 2C,D). A similar expression profile was observed in the ileum (Figure 2E,F). These findings suggest that iron deficiency did influence the differentiation pattern of ISCs. Besides differentiation, ISCs also self-renew and proliferate to maintain the intestinal epithelium. Iron deficiency significantly (*p* < 0.05) suppressed both mRNA and protein levels of Lgr5 (marker of ISCs) across the duodenum, jejunum, and ileum (Figure 2G,H). Consistent with this, Ki67 transcriptional level and the number of Ki67-positive proliferating cells were significantly (*p* < 0.05) lower in the iron-deficient group than those in the control group (Figure 2H,I). Taken together, our results indicate that iron deficiency not only biases ISCs differentiation toward enterocytes but also impairs their proliferative capacity.

### 3.3. Iron Is Essential for the Proliferation and Differentiation of ISCs in Enteroids

**Figure 3:**
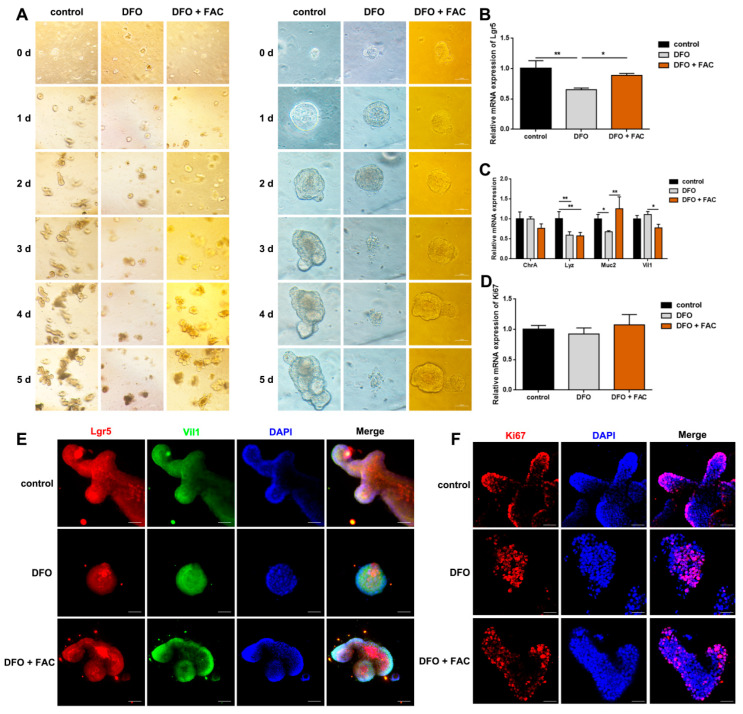
Iron is essential for the growth of intestinal stem cells in vitro. (**A**) Iron is essential for the growth of enteroids (Scale bar: 10 µm for **left panels**, 5 µm for **right panels**); (**B**) The relative mRNA expression of Lgr5 in enteroids (*n* = 5 per group); (**C**) The relative mRNA expression of ChrA, Lyz, Muc2 and Vil1 in enteroids (*n* = 5 per group); (**D**) The relative mRNA expression of Ki67 in enteroids (*n* = 5 per group); (**E**) Representative images of immunofluorescence staining with Lgr5 and Vil1 antibody of enteroids (Scale bar: 20 µm); (**F**) Representative images of immunofluorescence staining with Ki67 antibody of enteroids (Scale bar: 20 µm). Data are presented as mean ± SEM. Statistical significance was determined using Student’s *t* test. * *p* < 0.05, ** *p* < 0.01.

In order to verify the effect of iron deficiency on intestinal cells in vivo, we established an iron-deficient enteroid model by treating mouse enteroids with 500 μM deferoxamine (DFO). Compared with the control group, DFO-treated enteroids showed collapsed structure and cell death after 3 days, which was effectively rescued by supplementation with 500 μM ferric ammonium citrate (FAC) (Figure 3A). Based on these observations, we selected a 2-day treatment period for establishing the iron-deficient mouse enteroid model. DFO treatment significantly (*p* < 0.01) reduced the relative mRNA expression of *Lgr5*, which was reversed by FAC supplementation (Figure 3B). In addition, the mRNA levels of *Lyz* and *Muc2* was significantly (*p* < 0.05) decreased in DFO-treated enteroids compared with controls, and the reduction in *Muc2* transcript was restored by FAC treatment (Figure 3C). In contrast, the mRNA expression of *Ki67* remained unaffected by either DFO or FAC treatment (Figure 3D). Immunofluorescence analysis further confirmed these findings, showing consistent changes in Lgr5-positive ISCs, Vil1-positive cells and Ki67-positive proliferative cells (Figure 3E,F). These results align with the in vivo data, supporting the conclusion that iron deficiency inhibits the proliferation and differentiation of ISCs without affecting their proliferative capacity.

### 3.4. Iron Deficiency Disrupts ISC Self-Renewal and Enhances Enterocyte Differentiation by Activating Notch Signaling

**Figure 4:**
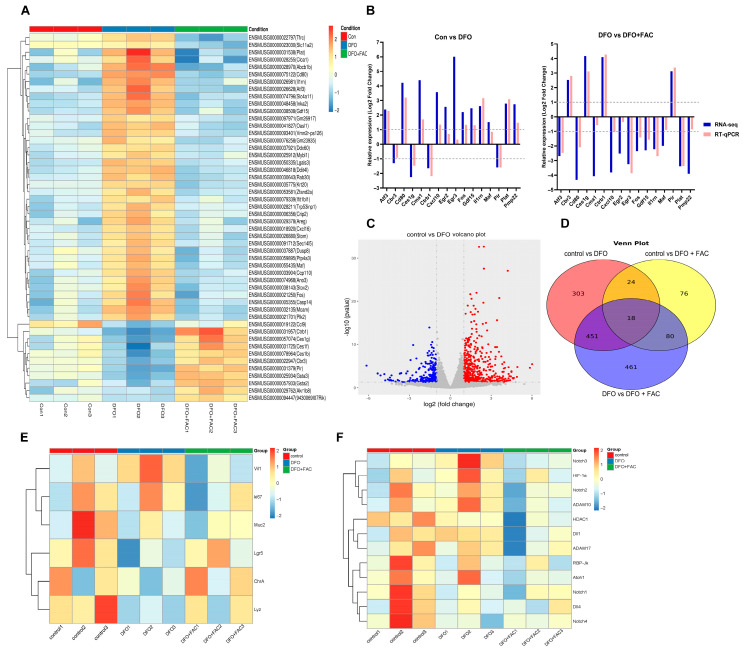
Iron deficiency enhances enterocyte differentiation by activating Notch signaling. (**A**) Cluster analysis of deferentially expressed genes (DEGs) (*n* = 3 per group, independent enteroid culture experiments); (**B**) Selected differentially expressed genes identified by RNA-seq were validated using qPCR. Expression levels (log_2_ fold change) from both methods showed strong concordance across the same samples (*n* = 4 per group, biological replicates; qPCR data are presented as mean ± SEM from 3 technical replicates per biological sample). (**C**) Volcano plot of DEGs between the control and DFO groups (significance thresholds: P_adj_ < 0.05 and |log_2_ fold change| > 0); (**D**) Venn diagram of DEGs among control vs. DFO, control vs. DFO + FAC, and DFO vs. DFO + FAC groups; (**E**,**F**) Heatmap showing relative expression of ISCs related markers and Notch signaling pathway related genes from DEGs (Only genes with *p* < 0.05 are shown).

To investigate how iron deficiency affects the proliferation and differentiation of ISCs, we performed RNA sequencing on mouse enteroids from control, DFO and DFO + FAC groups (Figure 4A). Following data filtration, quality control of sequencing error rates and GC content distribution, high-quality reads were obtained for downstream analysis. Differentially expressed genes (DEGs) were identified using the thresholds |log_2_FoldChange| > 0 and P_adj_ < 0.05. The reliability of the RNA-seq data was validated by RT-qPCR on 16 randomly selected DEGs, which showed consistent expression trends (Figure 4B). Detailed primer sequences and validation data are provided in Supplementary Figure S1. Volcano plot analysis revealed 796 DEGs between the control and DFO groups, with 528 upregulated and 286 downregulated genes (Figure 4C). Venn analysis indicated that 469 of these genes were reversibly regulated upon iron supplementation (Figure 4D). GO enrichment analysis showed that DEGs were significantly associated with biological processes (BP) such as cellular movement and motility; cellular components (CC) including the apical membrane and adherens junctions; and molecular functions (MF) related to receptor regulation and ligand activity (Figure S2A). These results suggest that iron deficiency disrupts epithelial organization and promotes cell migration, potentially impairing intestinal barrier integrity. KEGG pathway analysis further identified significant enrichment in apoptosis and endocrine resistance-related pathways (Figure S2B). Clustering of ISC-related markers demonstrated that DFO treatment significantly upregulated the enterocyte marker Vil1, while downregulating the ISC marker Lgr5 and secretory lineage markers (Muc2, ChrA, Lyz) (Figure 4E). These alterations were rescued by FAC, confirming that iron deficiency skews ISC differentiation toward the absorptive lineage. Notably, key components of the Notch signaling pathway were modulated under iron deficiency: the ligand *Dll1*, receptors *Notch2* and *Notch3*, and protease *ADAM10* were upregulated, whereas the negative regulator *Atoh1* was downregulated (Figure 4F). This suggests that Notch pathway modulation may contribute to enterocyte differentiation during iron deprivation, though further functional validation is needed.

## 4. Discussion

Iron deficiency is a prevalent nutritional disorder that often leads to intestinal malabsorption and mucosal damage [16]. However, current research lacks comprehensive studies on how iron deficiency affects ISC and their differentiation potential, with limited validation using animal and laboratory models [17]. This study elucidated the multifaceted impact of dietary iron deficiency on intestinal homeostasis, primarily through altering the fate determination of ISCs. By integrating in vivo mouse models and in vitro organoid systems, we demonstrated that iron deprivation disrupts the intestinal epithelial barrier via a dual mechanism: suppressing the self-renewal capacity of ISCs and skewing their lineage commitment, ultimately compromising tissue regeneration and integrity.

Our findings revealed a compartmentalized adaptation of the intestine to systemic iron deficiency. The duodenum exhibited a coordinated upregulation of transporters (TfR, FPN1) to enhance absorption, while the jejunum and ileum engage DMT1 and TfR. Paradoxically, this is accompanied by a systemic downregulation of iron storage (FTH), export (FPN1), and regulatory (HAMP, LF) proteins in distal segments, creating a maladaptive cycle that exacerbates iron retention dysfunction [12]. This dysregulated iron dynamics correlates with a significant reduction in ISC numbers and proliferative activity, as evidenced by decreased expression of the canonical stem cell marker Lgr5, coupled with a reduction in Ki67-positive proliferating cells. The association between nutrient deficiency and impaired ISC proliferation has been noted in other models of malnutrition, highlighting a potential common pathway in nutrient stress responses [18,19].

Crucially, beyond merely inhibiting proliferation, iron availability actively shaped differentiation fate. We observed a coordinated downregulation of key secretory lineage markers (ChrA, Lyz, Muc2) alongside a marked upregulation of the absorptive enterocyte marker Vil1. In addition, the iron chelator DFO also effectively recapitulated the phenotypes of dietary deficiency, confirming the central role of iron availability. DFO treatment reduced Lgr5+ ISCs, inhibited organoid growth, and mirrored the in vivo shift in differentiation markers. This “pro-absorptive, anti-secretory” shift was consistent across both experimental models, suggesting it is an intrinsic response to iron scarcity. This adaptive response may aim to maximize the absorptive surface area, albeit at the potential cost of compromising secretory functions critical for mucosal defense (e.g., antimicrobial peptide secretion by Paneth cells, mucus production by goblet cells) [20].

To explore the underlying mechanism, transcriptional profiling of iron-deficient enteroids was performed. RNA-seq analysis demonstrated that iron deficiency not only suppressed the proliferative capacity of ISCs (as indicated by downregulated *Lgr5* and *Ki67*) but, more importantly, biased their differentiation toward the absorptive enterocyte lineage at the expense of secretory lineages (e.g., Paneth cells, goblet cells) by activating the Notch signaling pathway (upregulating *Dll1*, *Notch2/3*, and downregulating *Atoh1*). The Notch pathway is a well-established arbiter of secretory-absorptive fate decisions in the gut [21]. Therefore, our data support a model wherein iron deficiency creates a cellular milieu that favors Notch activation, thereby directing ISCs toward the absorptive lineage while suppressing secretory cell differentiation. Mechanistically, iron chelation is known to stabilize HIF-1α, which may subsequently influence critical stem cell pathways such as Wnt/β-catenin. However, the precise hierarchical relationship and integration between iron-sensing mechanisms (HIF), key developmental pathways (Wnt/Notch), and the observed epigenetic regulators in driving the ISC phenotype remain unclear and represent a crucial area for future research using gene-editing approaches in advanced models.

The consequences of these cellular changes are profound for intestinal physiology. The depletion of Paneth (reduced Lyz) and goblet (reduced Muc2) cells directly weakens innate immune defense and mucus barrier integrity, respectively [20]. The potential impairment of enteroendocrine cell function (reduced ChrA) may disrupt hormonal signaling. This collective erosion of secretory lineage functions shares pathological similarities with conditions like inflammatory bowel disease (IBD), suggesting a plausible mechanistic link between chronic iron deficiency and increased susceptibility to intestinal inflammation. The disruption likely extends beyond mature cells, as the depletion of the Lgr5+ stem cell pool indicates a fundamental reprogramming of epithelial regeneration, possibly through iron-dependent epigenetic modifications affecting master differentiation regulators (e.g., Atoh1, Neurog3).

A limitation of this study is that the role of Notch signaling in iron deficiency-induced ISC differentiation is supported only by transcriptional data; we have not excluded the possibility that these changes are a compensatory response to other iron deficiency-related cellular events, and functional validation (e.g., using Notch inhibitors such as DAPT) will be necessary to confirm whether Notch signaling directly mediates the observed phenotype. Additionally, the sample size and experimental duration may constrain the detection of long-term adaptive effects of iron deficiency on ISC behavior and intestinal homeostasis. Importantly, our experimental design did not account for the gut microbiota—a well-established modulator of host iron metabolism, intestinal epithelial function, and stem cell niche regulation. The complex interplay between iron deficiency, microbial dysbiosis, and ISC proliferation/differentiation represents a critical frontier for future research, which will require integrated multi-omics approaches and organoid-microbe co-culture systems to dissect the underlying mechanisms.

## 5. Conclusions

In summary, iron deficiency perturbed intestinal homeostasis by directly targeting ISCs, impairing their self-renewal and biasing differentiation toward enterocytes at the expense of secretory lineages. This is mediated through a complex interplay of localized iron transport adaptations, systemic regulatory imbalances, and activation of Notch signaling pathway. Our findings underscore the essential role of iron in maintaining the intestinal stem cell niche and epithelial barrier function. From a translational perspective, these insights highlight that iron supplementation strategies for conditions like anemia should consider their reparative effects on the ISC compartment and mucosal regeneration, offering potential avenues for improving intestinal health and productivity in both clinical and agricultural settings. Future work should focus on dissecting the precise epigenetic and molecular mechanisms linking iron sensing to stem cell fate determination.

## Figures and Tables

**Table 1:** List of real time PCR primers and sequences.

Gene	Forward Primers	Reverse Primers	Primer Efficiency	R^2^
*ChrA*	CAGCTCGTCCACTCTTTCCG	AGGACGCACTTCATCACCTTG	93.2%	>0.999
*Ki67*	ACCATCATTGACCGCTCCTT	TCACTCTTGTCAGGGTCAGC	119.3%	>0.999
*Lgr5*	CACCCCAATGCGTTTTCTAC	GATGGTATCAGGCTCTGTAAGG	118.9%	>0.999
*Lyz*	AATGGATGGCTACCGTGGTGT	TAGTCGGTGCTTCGGTCTC	106.3%	>0.999
*Muc2*	GCCTGTTTGATAGCTGCTATGTGCC	GTTCCGCCAGTCAATGCAGACAC	110.1%	0.9997
*Vil1*	GCCAGATTGCTGACGAGGTT	GGCCCTAGTGAAGTCTTCGG	103.3%	0.9737
*β-actin*	GACGGCCAGGTCATCACTATTG	AGGAAGGCTGGAAAAGAGCC	91.7%	0.9867

**Table 2:** List of antibodies used in Western blot (WB) and immunofluorescence (IF) analysis.

Antibodies	Source	Identifier	Application
Anti-ChrA	Abcam (Cambridge, UK)	ab15160	WB, IF
Anti-Claudin1	HuaBio (Woburn, MA, USA)	HA721999	WB
Anti-DMT1	Abcam (Cambridge, UK)	ab55735	WB
Anti-FPN1	Abcam (Cambridge, UK)	ab239511	WB
Anti-FtH	Abcam (Cambridge, UK)	ab65080	WB
Anti-Ki67	Abcam (Cambridge, UK)	ab15580	IF
Anti-Lf	HuaBio (Woburn, MA, USA)	ER1912-16	WB
Anti-Lgr5	Abcam (Cambridge, UK)	ab75732	WB, IF
Anti-Lyz	Abcam (Cambridge, UK)	ab108508	WB, IF
Anti-Muc2	Diagbio (Guangzhou, China)	db16038	WB
Anti-Occludin	HuaBio (Woburn, MA, USA)	ET1701-76	WB
Anti-TfR	Abcam (Cambridge, UK)	ab84036	WB
Anti-Vil1	Proteintech (Rosemont, IL, USA)	16488-1-AP	WB, IF
Anti-ZO-1	HuaBio (Woburn, MA, USA)	HA722797	WB
Anti-β-actin	Abclonal (Woburn, MA, USA)	AC026	WB
Alexa Fluor 488 goat anti-rabbit	Abcam (Cambridge, UK)	ab150077	IF

## Data Availability

The data used to support the findings of this study are available from the corresponding author upon request.

## Supplementary Materials

The following supporting information can be downloaded at: https://www.mdpi.com/article/10.3390/nu18030392/s1, Figure S1: (A) The RNA-Seq analysis results of enteroids treated with DFO were verified by qPCR. The relative mRNA level of Atf3, Cbr3, Cd80, Ces1g, Cma1, Ctrb1, Cxcl10, Egr2, Egr3, Gdf15, Il1rn, Pir, Plat, Pmp22 and Stc1 coincided with RNA-Seq analysis; (B) Primer sequences used for qRT-PCR validation of RNA-seq data. Figure S2: (A) GO enrichment analysis; (B) KEGG pathway analysis.

## Abbreviations

The following abbreviations are used in this manuscript:

ISCs	Intestinal stem cells
DFO	Deferoxamine
ZO-1	Zonula Occludens-1
ChrA	Chromogranin A
Lyz	Lysozyme
Muc2	Mucin 2
Vil1	Villin 1
Lgr5	Leucine-rich repeat-containing G-protein coupled receptor 5
Dll1	Delta-like ligand 1
ADAM10	A Disintegrin And Metalloproteinase 10
Atoh1	Atonal Homolog 1
FPN1	Ferroportin 1
DMT1	Divalent metal transporter 1
TfR	Transferrin receptor
