# Supplementary material for: Iron Deficiency Inhibits the Proliferation of Intestinal Stem Cells and Induces Their Differentiation to Enterocytes
Source: Nutrients. 2026 Jan 24;18(3):392. doi: 10.3390/nu18030392 (PMC12899418; doi:10.3390/nu18030392)

Supplementary materials

Figure S1A. The RNA-Seq analysis results of enteroids treated with DFO were verified by qPCR. The relative mRNA level of *Atf3, Cbr3, Cd80, Ces1g, Cma1, Ctrb1, Cxcl10, Egr2, Egr3, Gdf15, Il1rn, Pir, Plat, Pmp22* and *Stc1* coincided with RNA-Seq analysis.

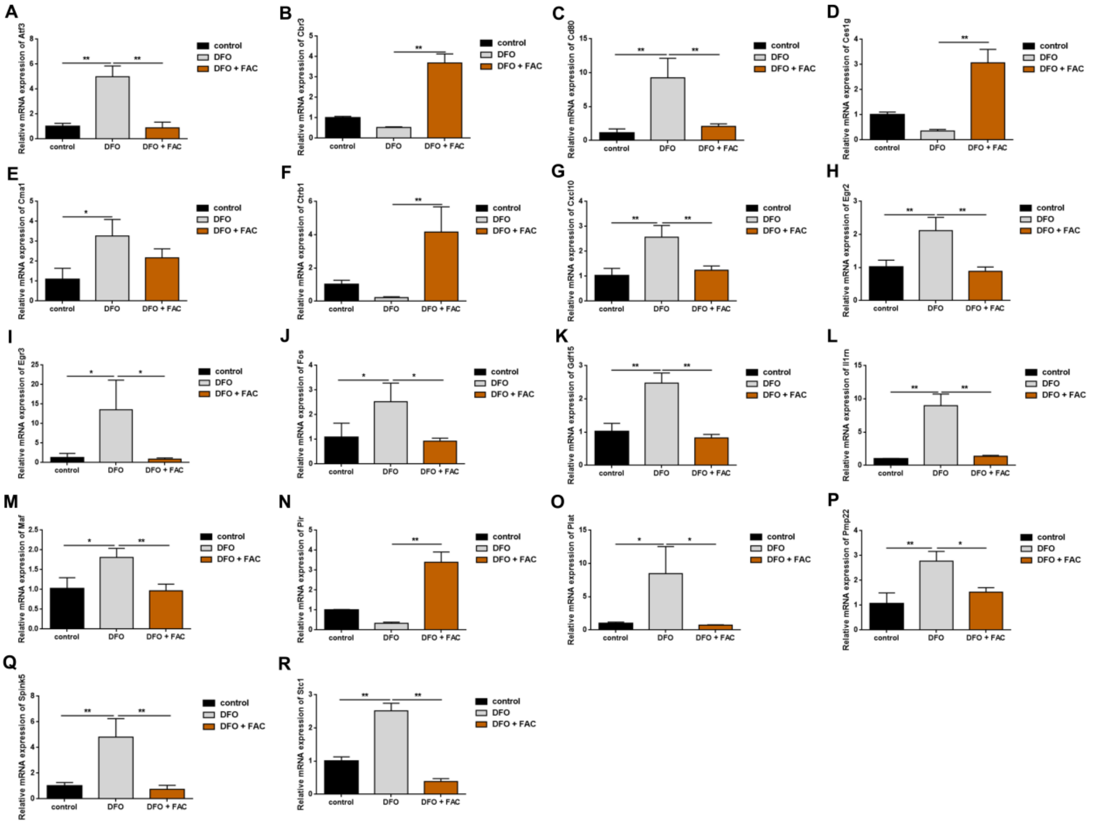

Figure S1B. Primer sequences used for qRT-PCR validation of RNA-seq data.

Gene	Forward primers	Reverse primers
Atf3	AGGCAGGAGCATCCTTTGTC	CTGCTTTGCATAGGACCCCA
Cbr3	CACATCAAGGGGGAAGGTCC	GAGAGTGTGGACACAGCGAA
Cd80	TGCCTTGCCGTTACAACTCT	GTATGTGCCCCGGTCTGAAA
Ces1g	GGCAGGCCTACCCAATTCTT	TCTGTCATTGTGGCAGGGTC
Cma1	CACGGAGTGCATACCACACT	CAGGCCGACAGGTAGTTCTC
Ctrb1	CAGGATCGTCAACGGAGAGG	ACATCGGTTGTCTTGACCCC
Cxcl10	ATGACGGGCCAGTGAGAATG	TCAACACGTGGGCAGGATAG
Egr2	GGTTGTGCGAGGAGCAAATG	GAAGATGGTCACCGACGAGGA
Egr3	TGACCGGAGGAGATGGTCTT	GTCCTGAGGCCTAAGAGGGA

Fos	GTTCGTGAAACACACCAGGC	GGCCTTGACTCACATGCTCT
Gdf15	CGACATGGCCCCGCC	GTTGAGTTGGGACTCAGGGC
Il1rn	GAAGGCAGTGGAAGACCTTGT	ATGAGCTGGTTGTTTCTCAGGT
Maf	TTCAAGAGGGTGCAGCAGAG	CGTTTTCTCGGAAGCCGTTG
Pir	GCAGTCAGAGGGGGTAGGA	CCTCGGTGTGGATGATCAGG
Plat	TCGGGACACAGAAGAAACGG	TTGTCTGCGTTGGCTCATCT
Pmp22	AACTGAAACAGGCACCGCT	GCCAGAGATCAGTCGTGTG
β-actin	GACGGCCAGGTCATCACTATTG	AGGAAGGCTGGAAAAGAGCC

Figure S2A GO enrichment analysis

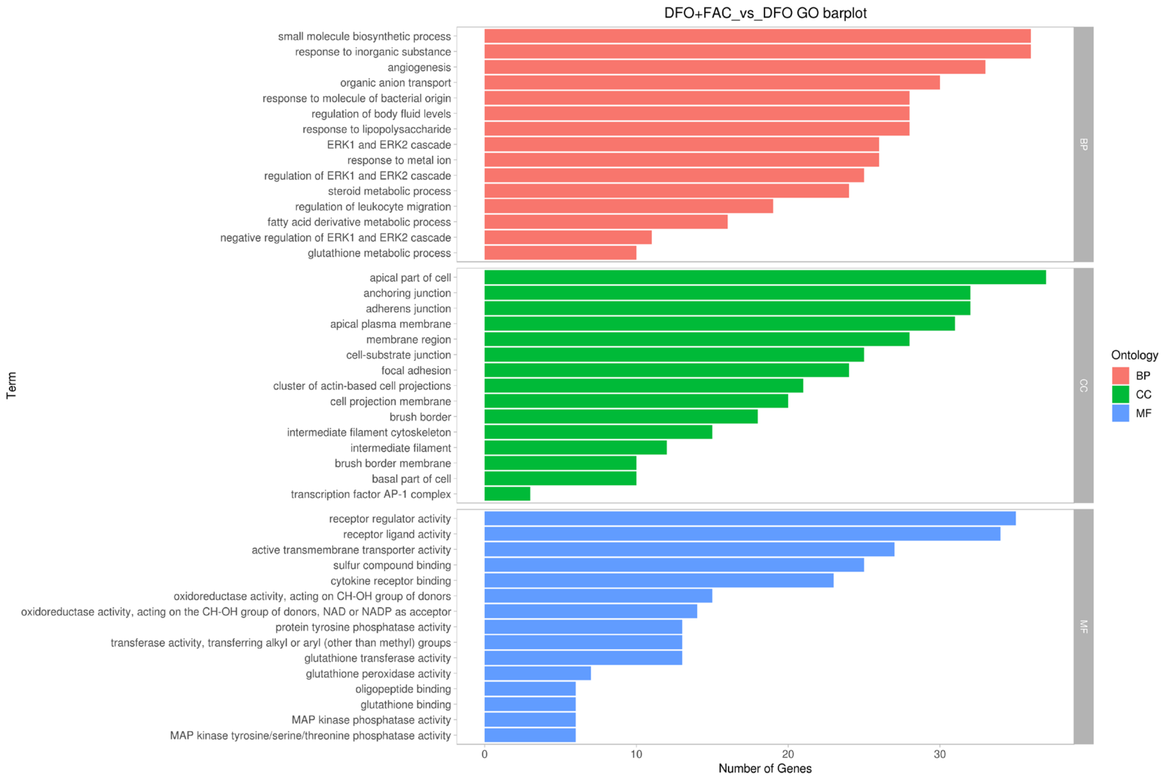
DFO+FAC_vs_DFO GO barplot
small molecule biosynthetic process
response to inorganic substance
angiogenesis
organic anion transport
response to molecule of bacterial origin
regulation of body fluid levels
response to lipopolysaccharide
ERK1 and ERK2 cascade
response to metal ion
regulation of ERK1 and ERK2 cascade
steroid metabolic process
regulation of leukocyte migration
fatty acid derivative metabolic process
negative regulation of ERK1 and ERK2 cascade
glutathione metabolic process
apical part of cell
anchoring junction
adherens junction
apical plasma membrane
membrane region
cell-substrate junction
focal adhesion
cluster of actin-based cell projections
cell projection membrane
brush border
intermediate filament cytoskeleton
intermediate filament
brush border membrane
basal part of cell
transcription factor AP-1 complex
receptor regulator activity
receptor ligand activity
active transmembrane transporter activity
sulfur compound binding
cytokine receptor binding
oxidoreductase activity, acting on CH-OH group of donors
oxidoreductase activity, acting on the CH-OH group of donors, NAD or NADP as acceptor
protein tyrosine phosphatase activity
transferase activity, transferring alkyl or aryl (other than methyl) groups
glutathione transferase activity
glutathione peroxidase activity
oligopeptide binding
glutathione binding
MAP kinase phosphatase activity
MAP kinase tyrosine/serine/threonine phosphatase activity
Term
BP
CC
MF
0
10
20
30
Number of Genes
Ontology
BP
CC
MF

Figure S2B KEGG pathway analysis

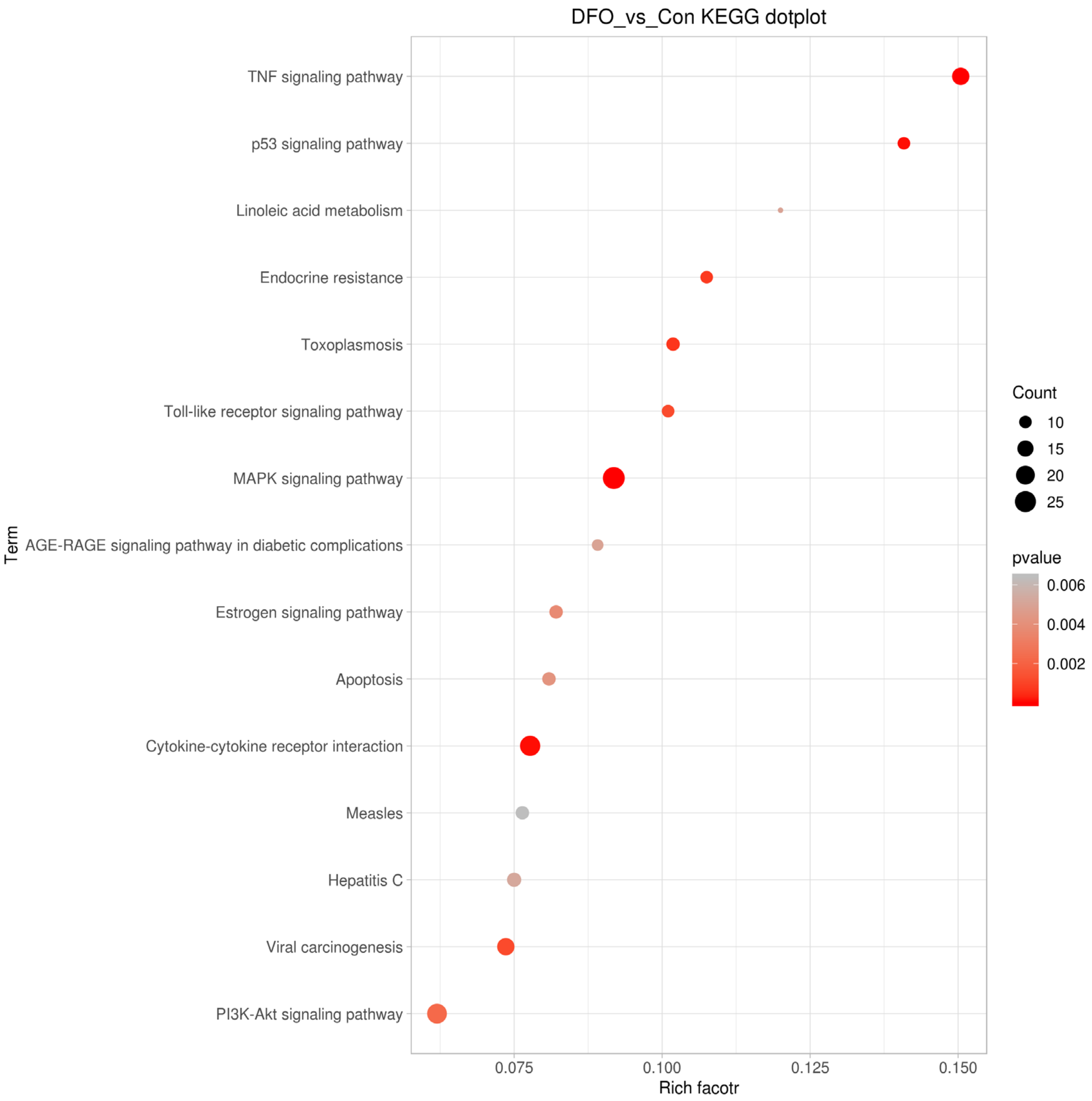

Supplement: Supplementary file 1 [file nutrients-18-00392-s001.zip › nutrients-4099936-supplementary.pdf]
